# Risk factors for skin tears in the older adults: a systematic review and meta-analysis

**DOI:** 10.3389/fpubh.2026.1880249

**Published:** 2026-08-03

**Authors:** Aixuan Guan, Yuxin Huang, Yating Guo, Huimin Huang, Jing Lv, Chaoqun Yang, Shi Wei, Bihong Chen

**Affiliations:** 1 Longyan First Affiliated Hospital of Fujian Medical University, Longyan, China; 2 Zhongshan Hospital Xiamen University, Xiamen, China; 3 Zhangzhou Municipal Hospital of Fujian Province, Zhangzhou, China

**Keywords:** elderly, factors, meta-analysis, nursing care, skin tears

## Abstract

**Background:**

A skin tear is a traumatic wound caused by mechanical forces, including removal of adhesives and patient handling, the depth of which may vary (not extending through the subcutaneous layer). With the global trend of an aging population, it has become a problem that requires great attention in clinical care. Previous studies have investigated the risk factors for ST in older adults (e.g., age, history of ST, malnutrition, etc.). However, the results of the studies varied widely, and the correlation coefficients of the results of different studies were also very different. To identify risk factors for the occurrence of ST in the old adults.

**Methods:**

We searched the following Chinese and English databases (PubMed, Web of Science, Cochrane Library, Embase, Medline, CINAHLA, Scopus, China Biomedical Database, Wanfang Database, and CNKI, VIP Database) to identify the risk factors for the occurrence of ST in the older adults related literature. The meta-analysis was conducted using Rev. Man 5.4.

**Results:**

A total of 10 articles (*n* = 6,206) were included in this analysis. The following risk factors were found to be significantly associated with ST in older adults: male sex (OR = 2.44, 95% CI [1.58, 3.78], *p* < 0.0001), having an Increased pressure ulcer risk (OR = 2.96, 95% CI [1.30, 6.74], *p* = 0.01), history of skin tears (OR = 9.34, 95% CI [1.52, 57.40], *p* = 0.02), and purpura (OR = 3.31, 95% CI [2.16, 5.07], *p* < 0.001).

**Conclusion:**

Many risk factors are associated with ST in the older adults. Nursing staff should emphasize the assessment of ST and improve prevention and care for those at risk.

**Systematic review registration:**

https://www.crd.york.ac.uk/PROSPERO/view/CRD42023479139.

## 1 Introduction

According to the International Skin Tear Advisory Panel (ISTAP) 2025 guidelines, a skin tear is defined as ‘a traumatic wound caused by mechanical forces, including removal of adhesives and patient handling, the depth of which may vary (not extending through the subcutaneous layer) (1). The main manifestations are the partial or complete stripping of the flap and the loss of the skin flap (2). The incidence of ST varies significantly across healthcare settings and patient populations, with a notably higher prevalence observed in elderly patients. This is due to a combination of internal factors, such as age-related skin changes (including thinning of the epidermis and increased fragility), and external factors, such as hydration status, how often a patient is bathed, and the use of medical adhesive products (3, 4). A meta-analysis indicates that the pooled prevalence of ST was 6.0%, and the pooled incidence was 11.0% (5). The prevalence of skin tears ranged from 1.06 to 22% in Australia, Belgium, Canada, China, Denmark, Japan and United States (6–13). With the increase in global aging, the older adults population is growing rapidly. According to the World Health Organization (14), between 2020 and 2030, the number of people over the aged over 60 years will increase from 1 billion to 1.4 billion, and one-sixth of the world’s population will be over the age of 60. ST, which is prone to poor healing and become chronic wounds, aggravates the condition or affects the quality of life, which in turn increases hospitalization costs, reduces the quality of life, causes physical and psychological pain to patients, and becomes an important issue affecting the safety of patients. Therefore, identification and prevention of risk factors are important to reduce the incidence of ST in the older adults.

Currently, the risk factors associated with the occurrence of ST in the older population are unclear. Although several studies on factors influencing older adults with ST have been undertaken, the risk factors studied were inconsistent and the conclusions were not consistent between studies. For example, Takeo et al. (15) reported that senile purpura, pseudoscar, contracture, and dry skin were identified as risk factors for ST in the people over 65, whereas age and history of skin tears were not associated with ST. However, LeBlanc et al. (16) found that history of an ST; the presence of skin changes associated with aging, ecchymosis, and hematomas; chronic disease; requiring assistance with activities of daily living; and displaying aggressive behavior were key risk factors associated with ST development. While there has been extensive research on risk factors for skin tears in older adults, there is currently less literature addressing quantitative synthesized. Only two previous systematic reviews (17, 18) have explored the risk factors for ST and their outcomes, but one of them only investigated the incidence of skin tears (17), the other systematic evaluation (18) only provided a descriptive summary of the included articles and did not use any statistical methods to integrate and analyze the results of the different articles. In addition, the most recent systematic evaluations of factors influencing skin tears was published in 2018, but many articles have been published in the last 2–3 years examining the risk factors associated with ST in older adults. For this reason, it is necessary to combine articles on the subject of risk factors for skin tears in the older adults with systematic quantitative evaluations. The aim of this systematic review was to synthesize the current evidence on risk factors for skin tears in the people over 65. Where sufficient homogeneity and data permitted, a meta-analysis was conducted to provide a quantitative summary of the effect sizes. It is important to note that a meta-analysis can only be conducted if the studies are sufficiently homogeneous, which is determined during the review process.

## 2 Methods

### 2.1 Study registration

This protocol for a systematic review and meta-analysis was pre-registered on the International Prospective Register of Systematic Reviews (PROSPERO), with the registration number CRD42023479139. The study followed the 2020 Preferred Reporting Items for Systematic Reviews and Meta-Analyses (PRISMA) standards (19).

### 2.2 Search strategy

We performed comprehensive searches of PubMed, Web of Science, Cochrane Library, Embase, Medline, CINAHL, Scopus, CNKI, Chinese biological medicine Database, Wanfang Database, VIP Database and retrospective searches of references included in the study. The selection and scope of these databases are summarized in Table 1. The search period was from the inception of the database to October 2023. In addition, study references were included retrospectively to supplement access to relevant literature. When we started searching, we used the following keywords: “skin tears” or “skin lacerations” and “risk factors” or “influence factors.” The search strategy for all databases is shown in the Supplementary Appendix.

**Table 1:** Database selection and content scope.

**Database**	Publisher/Host	Content scope
PubMed	National Library of Medicine (NLM), USA	Comprehensive biomedical literature and life sciences research
Web of Science	Clarivate Analytics	Multidisciplinary research covering sciences, social sciences, arts, and humanities
Cochrane Library	Cochrane Collaboration	Systematic reviews, clinical trials, and evidence-based healthcare research
Embase	Elsevier	Biomedical and pharmacological literature with extensive drug and medical device information
Medline	National Library of Medicine (NLM), USA	Biomedical literature including life sciences, behavioral sciences, and health services
CINAHL	EBSCO Information Services	Nursing, allied health, biomedical, and consumer health literature
Scopus	Elsevier	Comprehensive multidisciplinary abstract and citation database
CNKI (China National Knowledge Infrastructure)	Tsinghua Tongfang	Comprehensive academic resources in China covering all disciplines including medicine
Chinese Biomedical Literature Database (CBM)	Chinese Academy of Medical Sciences	Biomedical literature from China with extensive coverage of traditional Chinese medicine
Wanfang Database	Wanfang Data Co., Ltd.	Chinese scientific and technological literature including medical journals and dissertations
VIP Database	Chongqing VIP Information Co., Ltd.	Chinese scientific and technological journals with strong medical coverage

### 2.3 Inclusion and exclusion criteria

We adopted the Population, Intervention, Comparison, and Study Design (PICOS) framework (20) to serve as a guide for the development and establishment of eligibility criteria for this study. Studies had to meet the following criteria to be included in the systematic review and meta-analysis: (1) older adults patients (≥ 60 years) with ST; (2) it was available in English or Chinese;(3) Independent risk factors associated with ST are reported; (5) Study design included case–control studies, cohort studies, or cross-sectional studies; (6) The articles were analyzed using independent logistic regression, and the aOR /RR values were reported in the results of the studies.

Studies were excluded if: (1) the full articles could not be located; (2) relevant data were incomplete, inconsistent or could not be obtained after contacting the original authors. (3) The literature search was restricted to studies published between January 2015 and December 2022. This timeframe was chosen for two main reasons. The start date of 2015 accounted for the clinical translation lag following the International Skin Tear Advisory Panel (ISTAP) consensus published in 2011 (21), as the period from 2011 to 2014 represented a transitional phase in clinical practice. By beginning in 2015, we ensured the inclusion of studies that used fully established, standardized assessment protocols, such as the ISTAP classification system, thereby capturing the mature phase of guideline implementation rather than the variable early adoption period.

### 2.4 Quality of evidence assessment

We assessed the quality of the literature for case-cohort control studies and cohort studies using the Newcastle-Ottawa Scale (NOS) (22), which includes 3 categories of study population selection, between-group comparability, and measurement of outcome/exposure factors, with a total of 8 items and a total score of 9 points. A score of ≥7 was considered high quality, 4–6 as moderate quality, and 0–3 as low quality. Although the NOS scale is subject to ambiguity and excessive subjectivity, it remains one of the most widely used tools for evaluating the quality and risk of bias in observational studies (23). Cross-sectional studies were graded according to the Agency for Healthcare Research and Quality (AHRQ) (24) quality grading criteria and contained 11 items out of 11. A score of ≥8 was categorized as high quality, 4–7 as moderate quality, and 0–3 as low quality.

### 2.5 Study selection and risk of bias assessment

Following PRISMA recommendations (25), two researchers independently screened the titles/abstracts and full text of candidate articles based on inclusion/exclusion criteria. In case of disagreement, discussions were held with the concerned authors to reach consensus. Relevant data were extracted from the screened articles, including: first author, year of publication, country of study, type of study, sample size, and risk factors associated with the occurrence of ST in older adults. Two researchers independently assessed the risk of bias of the article using the Newcastle-Ottawa Scale (NOS) and the Agency for Healthcare Quality and Research (AHRQ) quality assessment criteria. A third author was consulted to resolve any disagreements.

### 2.6 Statistical analysis

Adjusted odds ratios (aORs) and 95% CIs were used for dichotomous variables. Meta-analysis was only performed when a potential risk factor was identified using Rev. Man 5.4 software in two or more studies. When statistical heterogeneity was low (*p* > 0.10; *I*^2^ ≤ 50%), a fixed-effects model was used. Otherwise, sensitivity and subgroup analyses were performed to explore potential sources of heterogeneity. If results remained heterogeneous, meta-analysis was performed using a random-effects model (26). To assess the stability of the results, a sensitivity analysis was performed by modifying the model. When more than 10 studies were included, a funnel plot and Begg and Egger tests were used to visually check for publication bias (27, 28). Descriptive analysis was used for risk factors in few studies or data that could not be pooled. *p* < 0.05 was considered statistically significant.

## 3 Results

### 3.1 Selection of studies

**Figure 1:**
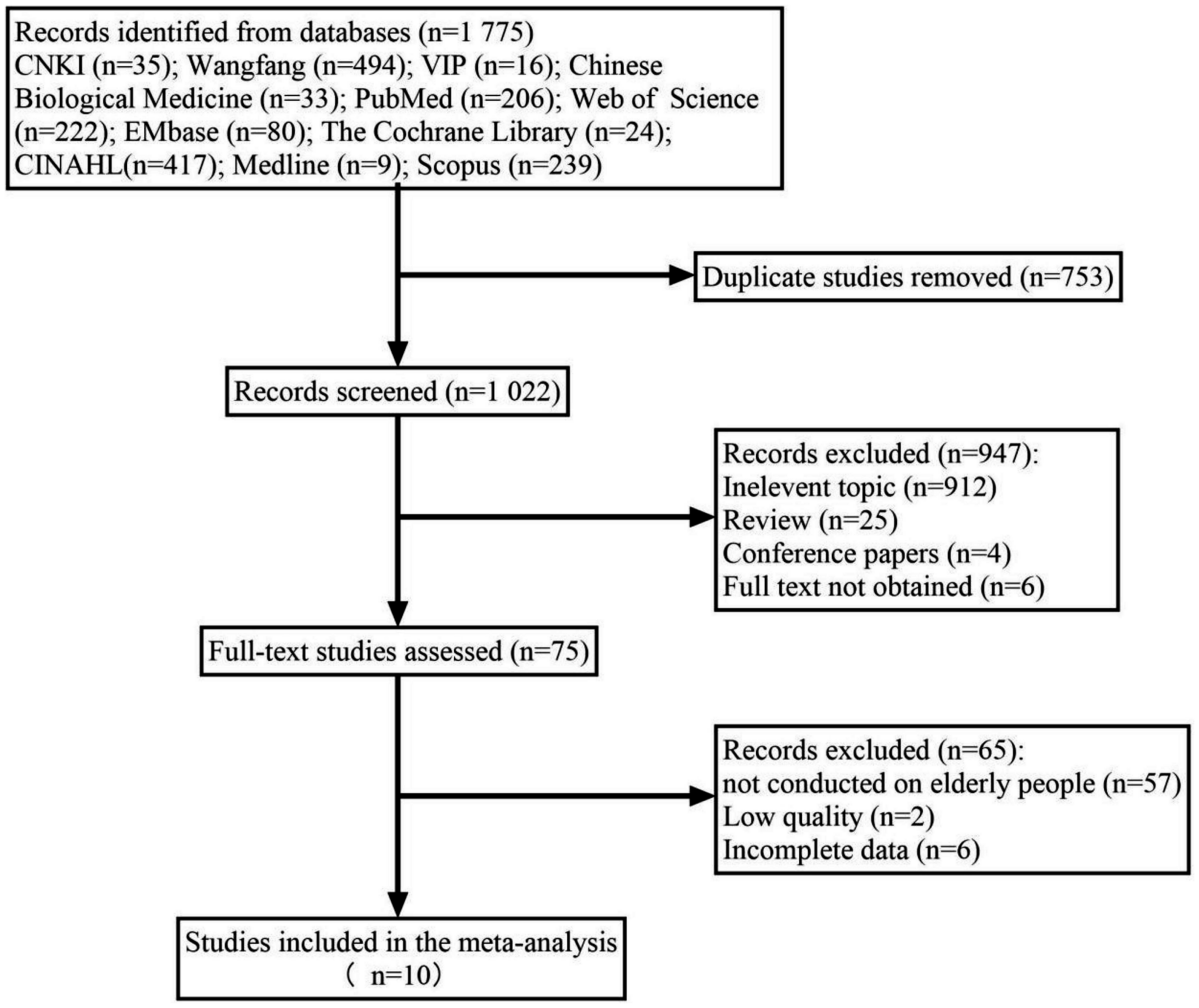
Literature selection flow chart.

A total of 1,775 articles, of which 753 were duplicates. After reading the titles and abstracts, we excluded 941 studies because they did not meet our eligibility criteria. After reading the full text, one case–control study (29), four cohort studies (15, 16, 30, 31), and five cross-sectional studies (6, 32–35) were eligible for inclusion in this meta-analysis. A total of 17,983 older adults were included, including 634 patients with skin tears, and 21 correlates were extracted. The prevalence of ST was in the range of 0.88 to 20.8% in all included studies. The quality of these studies was assessed as follows: case–control and cohort studies were graded on a scale of 6 to 8 using the NOS, and cross-sectional studies were graded on a scale of 6 to 9 using AHRQ-recommended grading criteria. The process used to select is shown in Figure 1. Detailed information on the inclusion of studies is in Table 2.

**Table 2:** Characteristics of the included studies.

First author	Year	Country	Study design	Total (*n*)	ST prevalence	Assessment tool	Study population characteristics	Significant factors	Quality evaluation
Qixia et al. (34)	2022	China	Cross-sectional study	14,675	0.88%	a	Mean age: 73.53 Range age: 60 to 102 Male: 8262 (56.3%) Female: 6413 (43.7%)	1, 2	9^②^
Qixia et al. (35)		China	cross-sectional study	14,470	0.89%	a	Mean age: 73.35 Range age: NA Male: 8180 (56.53%) Female: 6290 (43.47%)	2, 20	8^②^
Peres et al. (33)	2022	Brazil	cross-sectional study	69	11.6%	b	Mean age: 81 Range age: NA Male: 51 (73.9%) Female: 18 (26.1%)	3, 4	6^②^
LeBlanc et al. (16)	2021	Candana	cohort study	380	20.8%	c	Mean age: 85.4 Range age: 65 to 105 Male: 140 (36.8%) Female: 240 (63.2%)	1, 5, 6, 7, 8	7^①^
Minematsu et al. (15)	2021	Japan	cohort study	244	13.5%	b	Mean age: 88.1 Range age: NA Male: 64 (27.2%) Female: 180 (73.8%)	4, 9, 10	8^①^
Van Tiggelen et al. (32)	2019	Belgian	cross-sectional study	795	3.0%	a	Mean age: 85 Range age: NA Male: 247 (31.1%) Female: 548 (68.9%)	1, 6, 11, 12, 13	7^②^
Rayner et al. (31)	2019	Australia	cohort study	173	NA	b	Mean age: 87.6 Range age: NA Male: 50 (29%) Female: 123 (71%)	1, 4, 14, 15, 16	8^①^
Woo et al. (6)	2018	Candana	cross-sectional study	678	14.7%	c	Mean age: 75 Range age: 66 to 94 Male: 214 (31.6%) Female: 464 (68.4%)	2, 11,14	8^②^
Lewin et al. (29)	2016	Australia	case–control study	453	NA	b	Mean age: NA Range age: NA Gender: NA	1, 3, 4, 17, 18, 19	7^①^
Sanada et al. (30)	2015	Japan	cohort study	368	3.8%	b	Mean age: 87 Range age: NA Male: 94 (25.5%) Female: 274 (74.5%)	1, 2	7^①^

a: the International Skin Tear Advisory Panel (ISTAP) Classification System (2, 48), b: the Skin Tear Audit Research (STAR) Skin Tear Classification System (49), c: the Resident Assessment Instrument Minimum Data Set (RAI-MADS) (50), 1: history of skin tears, 2: having an increased pressure ulcer risk, 3: haematoma, 4: purpura, 5: skin changes associated with aging, ecchymosis, and hematomas, 6: activities of daily living, 7: chronic disease, 8: displaying aggressive behavior, 9: pseudoscar, 10:contracture, 11: age, 12: chronic use of corticosteroids, 13: use of adhesives/dressings, 14: male, 15: history of falls, 16: clinical manifestations of elastosis, 17: ecchymosis, 18:oedema, 19: inability to reposition oneself independently, 20: hypoproteinemia. ①: the Newcastle-Ottawa Scale; ②: the Agency for Healthcare Research and Quality quality grading criteria.

### 3.2 Risk factors

**Figure 2:**
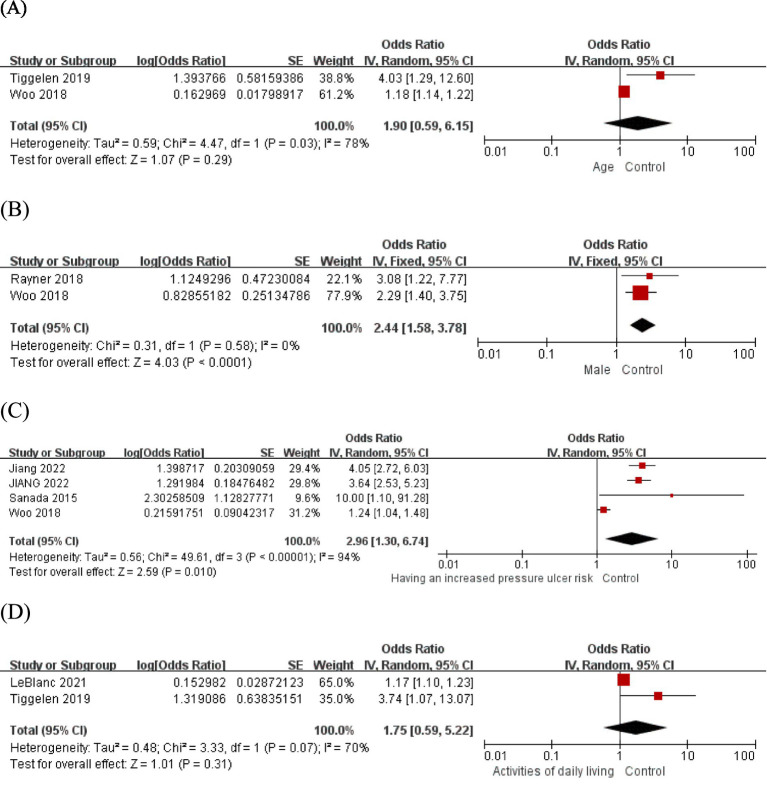
Forest of the meta-analysis **(A)** Age, **(B)** Male, **(C)** Having an increased pressure ulcer risk, **(D)** Activities of daily living.

Multivariate analysis identified a total of 22 risk factors in the 10 studies. Two stuies (15, 30) reported that two of these risk factors were not significantly associated with skin tears (Table 3). Subsequent meta-analyses, as described in Table 3, included 7 risk factors reported by at least two studies. Figure 2 shows the forest plot and sensitivity analysis results of the meta-analysis.

**Table 3:** Independent risk factors for ST in older adults in multivariable analyses.

Independent risk factors	Author (Year)	OR (95%CI)
History of skin tears	LeBlanc et al. (2021) (16)	2.29 (1.25, 4.20)
	Lewin et al. (2016) (16)	5.42 (4.83, 10.71)
	Rayner et al. (2019) (31)	3.82 (1.64, 8.90)
	Sanada et al. (2015) (30)	15.42 (3.53, 67.43)
	Van Tiggelen et al. (2019) (32)	3.83 (1.30, 11.32)
	Qixia et al. (2022) (34, 35)	230.24 (138.06, 383.98)
Having an increased pressure ulcer risk	Sanada et al. (2015) (30)	10.00 (1.10, 91.28)
	Woo et al. (2018) (6)	1.24 (1.04, 1.48)
	Qixia et al. (2022) (34, 35)	4.05 (2.72, 6.03)
	Qixia et al. (2022) (34, 35)	3.64 (2.53, 5.22)
Haematoma	Lewin et al. (2016) (29)	2.26 (1.30, 3.94)
	Peres et al. (2022) (33)	9.16 (1.52, 60.39)
Purpura	Lewin et al. (2016) (29)	2.66 (1.47, 4.81)
	Peres et al. (2022) (33)	6.27 (1.17, 39.24)
	Rayner et al. (2018) (31)	3.64 (1.42, 9.35)
	Takeo et al. (2021) (15)	4.28 (1.72, 10.65)
Skin changes associated with aging, ecchymosis, and hematomas	LeBlanc et al. (2021) (16)	1.86 (1.59, 2.19)
Activities of daily living	LeBlanc et al. (2021) (16)	1.17 (1.10, 1.23)
	Van Tiggelen et al. (2019) (32)	3.74 (1.09, 13.31)
Chronic disease	LeBlanc et al. (2021) (16)	1.22 (1.04, 1.43)
Displaying aggressive behavior	LeBlanc et al. (2021) (16)	1.08 (1.02, 1.13)
Pseudoscar	Takeo et al. (2021) (15)	2.67 (1.12, 6.39)
Contracture	Takeo et al. (2021) (15)	3.38 (1.18, 9.67)
Age	Van Tiggelen et al. (2019) (32)	4.03 (1.29, 12.61)
	Woo et al. (2018) (6)	1.18 (1.14, 1.22)
	Sanada et al. (2015) (30)	1.04 (0.96, 1.12)
	Takeo et al. (2021) (15)	1.05 (0.98, 1.11)
Chronic use of corticosteroids	Van Tiggelen et al. (2019) (32)	2.96 (1.06, 8.53)
Use of adhesives/dressings;	Van Tiggelen et al. (2019) (32)	7.05 (2.74, 18.14)
Male	Rayner et al. (2018) (31)	3.08 (1.22, 7.77)
	Woo et al. (2018) (6)	2.29 (1.40, 3.75)
History of falls	Rayner et al. (2018) (31)	3.37 (1.54, 7.41)
Clinical manifestations of elastosis	Rayner et al. (2018) (31)	3.19 (1.38, 7.38)
Ecchymosis	Lewin et al. (2016) (29)	6.24 (3.24, 12.01)
Oedema	Lewin et al. (2016) (29)	3.01 (1.62, 5.61)
Inability to reposition oneself independently	Lewin et al. (2016) (29)	2.31 (1.32, 4.04)
Hypoproteinemia	Qixia et al. (2022) (34, 35)	1.41 (1.01, 2.04)
Dry skin	Takeo et al. (2021) (15)	4.73 (1.00, 22.50)
Use of steroids	Sanada et al. (2015) (30)	6.31 (0.90, 44.18)

CI, confidence interval; OR, odds ratio.

#### 3.2.1 Effect of general factors on ST

In this study, general factors included age, gender, pressure ulcer risk score, and ability of daily life. Male gender was found to be significantly associated with the risk of ST in older adults in the meta-analysis of data from two studies (6, 31) (OR = 2.44, 95% CI [1.58, 3.78], *p* < 0.0001; *I*^2^ = 0%, *p* = 0.58). Four studies (6, 30, 34, 35) reported a statistically significant association between having an Increased pressure ulcer risk and ST in older adults (OR = 2.96, 95% CI [1.30, 6.74], *p* = 0.01; *I*^2^ = 94%, *p* < 0.0001) were significant risk factors for ST in older people. Sensitivity analysis by removing literature one at a time revealed Woo et al. (6) as a source of heterogeneity, and removing this literature reduced *I*^2^ from 94 to 0%. Statistical significance was confirmed in a pooled analysis of the remaining literature using a fixed effects model (OR = 3.87, 95% CI [2.97, 5.05], *p* = 0.01; *I*^2^ = 0%, *p* = 0.65). Although four studies (6, 15, 30, 32) performed logistic regression analyses of age and incident ST in older adults, only two studies found age to be a risk factor for incident ST in older adults, and a meta-analysis of these two studies (6, 32) found that age was not significantly associated with the occurrence of ST in older adults (OR = 1.90, 95% CI [0.59, 6.15], *p* = 0.29; *I*^2^ = 78%, *p* = 0.03). Pooled analysis of these two studies (16, 32) showed that activities of daily living scores did not significantly increase the prevalence of ST in older adults (OR = 1.75, 95% CI [0.59, 5.22], *p* = 0.31; *I*^2^ = 70%, *p* = 0.07). The forest plots of the meta-analysis are presented in Figure 2. The main outcomes of the meta-analysis are shown in Table 4.

**Table 4:** The main outcome of the meta-analysis.

Significant factors	Independent risk factors	No. of studies	Total sample	OR (95% CI)	*p* value	Heterogeneity of study design			Effect size model
						Chi^2^	*I*^2^ (%)	*Q*-test (*p*)	
General factors	Age	2	1,473	1.90 (0.59, 6.15)	0.29	4.47	78	0.03	Random
	Male	2	851	2.44 (1.58, 3.78)	<0.0001	0.31	0	0.58	Fixed
	Having an Increased pressure ulcer risk	4	15,721	2.96 (1.30, 6.74)	0.01	49.61	94	<0.0001	Random
	Activities of daily living	2	1,175	1.75 (0.59, 5.22)	0.31	3.33	70	0.07	Random
Skin disease factors	History of skin tears	6	16,844	9.34 (1.52, 57.40)	0.02	170.84	97	<0.0001	Random
	Hematoma	2	522	3.41 (0.98, 11.93)	0.05	2.03	51	0.15	Random
	Purpura	4	939	3.31 (2.61, 5.07)	<0.0001	1.38	0	0.71	Fixed

#### 3.2.2 Effect of skin factors on ST

**Figure 3:**
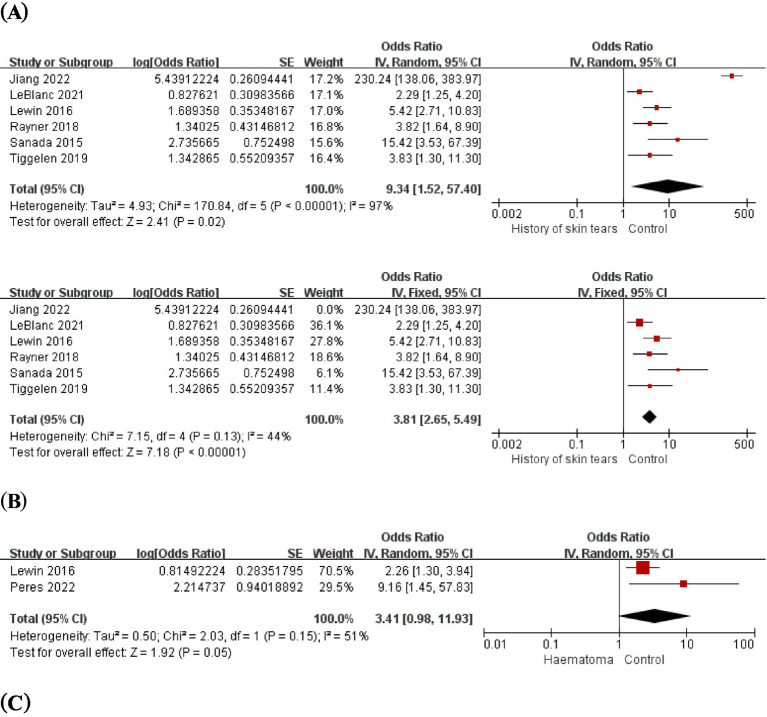
Forest of the meta-analysis: **(A)** History of skin tears, **(B)** hematoma, **(C)** purpura.

In this study, skin disease factors included history of skin tears, hematoma and purpura. The relationship between history of skin tears and ST in older population was explored in six studies (16, 29–32, 34), the results of which (OR = 9.34, 95% CI [1.52, 57.40], *p* = 0.02) were found to be highly heterogeneous (*I*^2^ = 97%, *p* < 0.0001). A sensitivity analysis was performed using case-by-case exclusion, and the article by Qixia et al. (34). was found to be a source of heterogeneity, which was reduced to a low level when this article was excluded. Therefore, after excluding this article, meta-analyses of the remaining studies using a fixed-effects model confirmed that their findings were statistically significant (OR = 3.81, 95% CI [2.65, 5.49], *p* < 0.00001; *I*^2^ = 44%, *p* = 0.13). Purpura was found to have been significantly associated with the risk of ST in older people in the meta-analysis of data from four studies (15, 29, 31, 33) (OR = 3.31, 95% CI [2.16, 5.07], *p* < 0.001; *I*^2^ = 0%, *p* = 0.71). No statistically significant difference was observed for hematoma (OR = 3.41, 95% CI [0.98, 11.93], *p* = 0.05; *I*^2^ = 51%, *p* = 0.15) by two studies (29, 33). The forest plots of the meta-analysis are presented in Figure 3. The main outcomes of the meta-analysis are shown in Table 4.

#### 3.2.3 Other relevant risk factors on ST

Skin changes associated with aging (16), ecchymosis, and hematomas; chronic disease (16); displaying aggressive behavior (16); pseudoscar (15); contracture (15); chronic use of corticosteroids (32); use of adhesives/dressings (32); history of falls (31); clinical manifestations of elastosis (31); ecchymosis (29); oedema (29); inability to reposition oneself independently (29); hypoproteinemia (35) were the other 13 individual risk factors identified via multiple regression analysis. These factors were not included in the meta-analysis due to insufficient data (i.e., identified in fewer than two studies), preventing statistical pooling.

## 4 Discussion

Here, we included 10 articles and 17,983 participants and identified a total of 4 risk factors for skin tears in older people via our meta-analysis. History of skin tears and purpura were found to have been strong risk factors (OR: 3.31–9.34); male sex and having an increased pressure ulcer risk were found to have been moderate risk factors (OR: 2.44–2.96). Our findings provide stronger and more appropriate evidence to identify and assess risk factors for the occurrence of ST in the older adults.

In our meta-analysis, chronological age was not identified as a statistically significant risk factor for skin tears in older adults. This finding appears to contradict previous evidence, such as the systematic review by Serra et al. (18), which identified age as a key risk factor. However, this discrepancy requires careful interpretation and likely reflects methodological limitations rather than a lack of biological association. First, our subgroup analysis included only two studies focusing specifically on older populations (*n* = 2), which substantially limited our ability to detect an effect compared to broader adult samples. Second, and more importantly, chronological age must be distinguished from the biological ageing of the skin. As El Genedy-Kalyoncu et al. (36) have noted, the pathophysiology of skin tears is undeniably linked to senescent changes—such as epidermal thinning, loss of dermal elasticity, and increased fragility—rather than the number of years lived per se. Since skin ageing is nearly universal among the older adults, isolating chronological age as an independent variable remains statistically challenging. Thus, although the statistical analysis did not identify age as a predictor, this should not be interpreted as evidence that age is irrelevant. Instead, it suggests that clinical assessments should prioritize biological markers of skin integrity (e.g., the presence of senile purpura or a history of skin tears) over chronological age alone. This perspective aligns with recent ISTAP (2025) (37) recommendations, which emphasize comprehensive skin assessment rather than age-based screening. Further research is needed to explore how these age-related morphological changes interact with extrinsic risk factors in different clinical settings.

In this meta-analysis, ST was associated with the general condition of the older adults. We found that older men were 2.44 times more likely to develop ST compared to women. This may be due to the fact that men are exposed to more UV light in their professional and recreational lives than women, and that prolonged exposure of the skin to UV light promotes vitamin D synthesis, leads to degenerative changes in the skin, and affects the cellular, fibrous, and vascular structure of the skin (38, 39). It is recommended that nurses increase health education on sun protection for male patients, emphasizing that sun protection is not merely a cosmetic concern but a crucial preventive measure for maintaining skin health and reducing the risk of skin tears.

In addition, the results of this study showed that the risk of ST was 2.96 times higher in older adults at risk for pressure ulcers than in patients not at risk for pressure ulcers. Although pressure ulcers and skin tears arise from different pathological mechanisms and commonly occur at different anatomical sites, they share several intrinsic risk factors, including impaired mobility, exposure to shear and friction forces, and reduced skin integrity. Therefore, an increased pressure ulcer risk score may reflect a broader vulnerability to skin injury rather than a direct causal relationship with ST. Forest plot and sensitivity analyses identified one study (6) to have been the primary cause of heterogeneity. As shown in Figure 2C, after the exclusion of this study, *post hoc* analyses showed having an increased pressure ulcer risk remained an important factor in the occurrence of ST in older adults (OR = 3.87, 95% CI [2.97, 5.05], *p* < 0.00001; *I*^2^ = 0%, *p* = 0.65). The differences in the Woo et al. study (6) compared to other studies may be due to the different pressure ulcer assessment tools used. The assessment tool used by Woo et al. was PURS (pressure ulcer risk score), which is one of the instruments found within the RAI-MDS, provides an estimate of graded risk for developing a pressure ulcer among individuals who did not present with a pressure ulcer at the time of assessment (40). While the other three studies used the “gold standard” (Braden Scale) for the assessment of pressure ulcers. Although the PURS has been validated and shows equal predictive ability when compared to the Braden Scale (41), differences still exist due to differences in scale items and score ranges. A previous study (11) has found that the prevalence of ST in elder people is similar to the prevalence of pressure ulcers (10.5% for skin tears and 8% for pressure ulcers), a finding that may indicate an association between ST and pressure ulcers. Having an Increased pressure ulcer risk means that the patient has endogenous and exogenous factors for the development of a pressure injury, including perception, wetness, mobility, friction and shear, and mobility (42), the combination of internal and external factors not only increases the risk of pressure ulcers, but may also increase the risk of ST in older adults. It is recommended that caregivers focus on older adults with Braden scores ≤16 who are at risk for pressure ulcers, assess their skin condition in a timely manner, promote turning, and increase awareness of how older adults can manage their own skin condition to minimize the occurrence of pressure ulcers and ST. A systematic review (43) found that emollients may be protective in maintaining the integrity of older skin, making it less susceptible to shear and friction.

Among the factors associated with skin disease, our review showed that a history of skin tears and purpura was associated with an increased risk of ST in the older adults. In this study, the risk of ST in older adults with a history of skin tears was 9.34 times higher than in those without a history of ST. However, the result had a significance heterogeneity. Forest plot and sensitivity analyses identified one study (34) to have been the primary cause of heterogeneity. As shown in Figure 3 (E), after the exclusion of this study, meta-analyses showed history of skin tears remained an important factor in the occurrence of ST in older population (OR = 3.81, 95% CI [2.65, 5.49], *p* < 0.00001; *I*^2^ = 44%, *p* = 0.13). The discrepancy in Qixia’s study (34) may stem from a much lower prevalence of ST among older individuals compared to other studies. Moreover, despite notable heterogeneity, Qixia’s study carried the highest weight in the meta-analysis and yielded results comparable to those of two other similarly weighted studies (16, 29). The inclusion of a history of skin tears as a variable in Qixia’s analysis rendered the previously negligible findings less robust (34). A history of skin tears increases the likelihood of recurrent ST in older adults, possibly due to the reduced tensile strength of scar tissue (44), it may also be related to the fact that patients with a history of ST are prone to recurrent ST as a result of disease or the effects of external injury. Therefore, for older adults with a history of skin tears, nurses should inquire about the time and location of the skin tear, check the skin condition, and record it in a timely manner so that the skin can be monitored dynamically.

Our study found that older population patients with purpura were 3.31 times more likely to develop ST than patients without purpura. Purpura commonly presents as noninflammatory, purplish, petechial lesions on the skin, typically measuring 2–20 mm in diameter and appearing flat or slightly elevated (45, 46). These lesions are often associated with skin aging and increased vascular fragility (47). The appearance of purpura indicates thinning of the skin at the site and its loss of protection or cushioning, and fragile blood vessels and frequent trauma increase the risk of ST. Nursing staff are advised pay close attention to older adults with skin purpura, improve their skin care, be aware of their coagulation mechanism, and work with physicians to identify and treat the etiology that triggers the development of purpura in older adults in order to reduce the risk of ST in older adults.

Several limitations should be considered regarding the studies included in this meta-analysis. For instance, the case–control study by Lewin et al. (29) included participants aged over 50 years. Similarly, the cohort study by Sanada et al. (30) identified only 14 patients with skin tears, which may limit the stability of its estimates. Rayner et al. (31) reported that a decreased Braden Scale score—a well-established pressure ulcer risk indicator—was not associated with skin tears, suggesting that the relationship between pressure injury risk and skin tears warrants cautious interpretation. These factors may contribute to the heterogeneity observed in our analysis and highlight the need for future high-quality, adequately powered prospective studies.

This meta-analysis suggests that increasing age, activities of daily living and hematoma are not risk factors for ST. This may be related to the small sample size in the literature. More studies are needed in the future.

The limitations of this meta-analysis need to be considered: (1) we only included observational studies in English or Chinese, which may be a source of publication bias; (2) the reliability of the review results may be affected by the fact that some risk factors were included in fewer studies; (3) some risk factor indicators in this meta-analysis were not effectively combined due to the limited amount of literature, which may have influenced the results; (4) our search was restricted to literature published between 2015 and 2022, which may have excluded relevant earlier studies.

## 5 Conclusion

In conclusion, this meta-analysis showed that being male, having an increased risk of pressure ulcer, history of skin tear and purpura were risk factors for ST in the older adults. This can be used as a basis for a clinical preventive management. More large-sample, multicenter, high-quality prospective studies are needed in the future to further identify the risk factors for the development of ST, which will help to develop appropriate preventive and interventional measures to reduce the incidence of ST in the older population.

## Data Availability

The original contributions presented in the study are included in the article/Supplementary material, further inquiries can be directed to the corresponding authors.
