# Supplementary material for: Risk factors for skin tears in the older adults: a systematic review and meta-analysis
Source: Front Public Health. 2026 Aug 3;14:1880249. doi: 10.3389/fpubh.2026.1880249 (PMC13478709; doi:10.3389/fpubh.2026.1880249)
Supplement: Supplementary file 1 [file Table_1.DOCX]

**SUPPLEMENTARY MATERIALS**

Skin tears and risk factors in the elderly: a systematic review and meta-analysis

Aixuan Guan^1^, Yuxin Huang ^2^, Yating Guo ^3^, Huimin Huang ^2^, Jing Lv^1^, Chaoqun Yang^1^, Shi Wei^1^, Bihong Chen^1^

(1 Department of Nursing, Longyan

First Hospital Affiliated to Fujian Medical University , Longyan, 36400,China;

2 Department of Nursing, Zhongshan Hospital Affiliated to Xiamen University, Xiamen 361004, China;

3 Department of Nursing, Zhangzhou First Hospital, Zhangzhou, 363099, China)

**S1 Table. Search strategy**

| **Database** | **Keyword** |
| --- | --- |
| **PubMed** | #1 “skin tears” [All Fields] OR “skin tear” [All Fields] OR “skin laceration” [All Fields] OR “skin lacerations” [All Fields]  #2 “risk factors” [MeSH Terms] OR “Factor, Risk” [All Fields] OR “risk factor” [All Fields] OR “Social Risk Factors” [All Fields] OR “Factor, Social Risk” [All Fields] OR “Factors, Social Risk” [All Fields] OR “Risk Factor, Social” [All Fields] OR “Risk Factors, Social” [All Fields] OR “Social Risk Factor” [All Fields] OR “Health Correlates” [All Fields] OR “Correlates, Health” [All Fields] OR “Population at Risk” [All Fields] OR “Populations at Risk” [All Fields] OR “Risk Scores” [All Fields] OR “factor” [All Fields] OR “influence factors” [All Fields]  #3 #1 AND #2 |
| **Web of Science** | #1 All Fields = (“skin tears” OR “skin tear” OR “skin laceration” OR “skin lacerations”)  #2 All Fields = (“risk factors” OR “Factor, Risk” OR “risk factor” OR “Social Risk Factors” OR “Factor, Social Risk” OR “Factors, Social Risk” OR “Risk Factor, Social” OR “Risk Factors, Social” OR “Social Risk Factor” OR “Health Correlates” OR “Correlates, Health” OR “Population at Risk” OR “Populations at Risk” OR “Risk Scores” OR “factor” OR “influence factors”)  #3 #1 AND #2 |
| **Scopus** | #1 TITLE-ABS-KEY (“skin tears” OR “skin tear” OR “skin laceration” OR “skin lacerations”)  #2 TITLE-ABS-KEY (“risk factors” OR “Factor, Risk” OR “risk factor” OR “Social Risk Factors” OR “Factor, Social Risk” OR “Factors, Social Risk” OR “Risk Factor, Social” OR “Risk Factors, Social” OR “Social Risk Factor” OR “Health Correlates” OR “Correlates, Health” OR “Population at Risk” OR “Populations at Risk” OR “Risk Scores” OR “factor” OR “influence factors”)  #3 #1 AND #2 |
| **Embase** | #1 All Fields = (“skin tears” OR “skin tear” OR “skin laceration” OR “skin lacerations”)  #2 All Fields = (“risk factors” OR “Factor, Risk” OR “risk factor” OR “Social Risk Factors” OR “Factor, Social Risk” OR “Factors, Social Risk” OR “Risk Factor, Social” OR “Risk Factors, Social” OR “Social Risk Factor” OR “Health Correlates” OR “Correlates, Health” OR “Population at Risk” OR “Populations at Risk” OR “Risk Scores” OR “factor” OR “influence factors”)  #3 #1 AND #2 |
| **Cochrane** | #1 Title Abstract Keyword (“skin tears” OR “skin tear” OR “skin laceration” OR “skin lacerations”)  #3 Title Abstract Keyword (“risk factors” OR “Factor, Risk” OR “risk factor” OR “Social Risk Factors” OR “Factor, Social Risk” OR “Factors, Social Risk” OR “Risk Factor, Social” OR “Risk Factors, Social” OR “Social Risk Factor” OR “Health Correlates” OR “Correlates, Health” OR “Population at Risk” OR “Populations at Risk” OR “Risk Scores” OR “factor” OR “influence factors”)  #3 #1 AND #2 |
| **Medline** | #1 All Fields = (“skin tears” OR “skin tear” OR “skin laceration” OR “skin lacerations”)  #2 All Fields = (“risk factors” OR “Factor, Risk” OR “risk factor” OR “Social Risk Factors” OR “Factor, Social Risk” OR “Factors, Social Risk” OR “Risk Factor, Social” OR “Risk Factors, Social” OR “Social Risk Factor” OR “Health Correlates” OR “Correlates, Health” OR “Population at Risk” OR “Populations at Risk” OR “Risk Scores” OR “factor” OR “influence factors”)  #3 #1 AND #2 |
| **CINAHL** | #1 (MM “skin tears”) OR “skin tear” OR “skin laceration” OR “skin lacerations”  #2 (MM “risk factors”) “Factor, Risk” OR “risk factor” OR “Social Risk Factors” OR “Factor, Social Risk” OR “Factors, Social Risk” OR “Risk Factor, Social” OR “Risk Factors, Social” OR “Social Risk Factor” OR “Health Correlates” OR “Correlates, Health” OR “Population at Risk” OR “Populations at Risk” OR “Risk Scores” OR “factor” OR “influence factors”  #3 #1 AND #2 |
| **CNKI** | #1 (主题=皮肤撕裂伤+皮肤撕裂+皮肤裂伤+皮肤破损+皮肤损伤)  #2 (主题=危险因素+影响因素+风险因素+相关因素)  #3 #1 AND #2 |
| **Chinese biological medicine Database** | #1 (常用字段=皮肤撕裂伤 OR皮肤撕裂OR皮肤裂伤OR皮肤破损OR皮肤损伤)  #2 (常用字段=危险因素OR影响因素OR风险因素OR相关因素)  #3 #1 AND #2 |
| **Wanfang Database** | #1 (主题=皮肤撕裂伤 OR皮肤撕裂OR皮肤裂伤OR皮肤破损OR皮肤损伤)  #2 (主题=危险因素OR影响因素OR风险因素OR相关因素)  #3 #1 AND #2 |
| **VIP Database** | 1 (题名或关键词=皮肤撕裂伤 OR皮肤撕裂OR皮肤裂伤OR皮肤破损OR皮肤损伤)  #2 (题名或关键词=危险因素OR影响因素OR风险因素OR相关因素)  #3 #1 AND #2 |
